# Diagnostic Accuracy of Obstructive Airway Adult Test for Diagnosis of Obstructive Sleep Apnea

**DOI:** 10.1155/2015/915185

**Published:** 2015-10-08

**Authors:** Giulio Gasparini, Claudio Vicini, Michele De Benedetto, Fabrizio Salamanca, Giovanni Sorrenti, Mario Romandini, Marcello Bosi, Gianmarco Saponaro, Enrico Foresta, Andreina Laforì, Giuseppe Meccariello, Alessandro Bianchi, Domenico Maurizio Toraldo, Aldo Campanini, Filippo Montevecchi, Grazia Rizzotto, Daniele Cervelli, Alessandro Moro, Michele Arigliani, Riccardo Gobbi, Sandro Pelo

**Affiliations:** ^1^Maxillo Facial Surgery, Complesso Integrato Columbus, Catholic University Medical School, Rome, Italy; ^2^Special Surgery Department, Ear-Nose-Throat Unit, Morgagni-Pierantoni Hospital, Forlì, Italy; ^3^Department of Otolaryngology Head and Neck Surgery, Hospital Fazzi, Lecce, Italy; ^4^Department of Otolaryngology, S. Pio X Hospital, Milan, Italy; ^5^Department of Otolaryngology Head and Neck Surgery, S. Orsola-Malpighi University Hospital, Bologna, Italy; ^6^Department of Medical, Oral and Biotechnological Sciences, University of Chieti-Pescara, Chieti, Italy; ^7^Department of Periodontology, Catholic University of the Sacred Heart, Rome, Italy; ^8^“A. Galateo” Respiratory Rehabilitation Unit, San Cesario di Lecce, Italy; ^9^Department of Neurology, Complesso Integrato Columbus, Rome, Italy

## Abstract

*Rationale. *The gold standard for the diagnosis of Obstructive Sleep Apnea (OSA) is polysomnography, whose access is however reduced by costs and limited availability, so that additional diagnostic tests are needed.* Objectives. *To analyze the diagnostic accuracy of the Obstructive Airway Adult Test (OAAT) compared to polysomnography for the diagnosis of OSA in adult patients.* Methods. *Ninety patients affected by OSA verified with polysomnography (AHI ≥ 5) and ten healthy patients, randomly selected, were included and all were interviewed by one blind examiner with OAAT questions.* Measurements and Main Results. *The Spearman rho, evaluated to measure the correlation between OAAT and polysomnography, was 0.72 (*p* < 0.01). The area under the ROC curve (95% CI) was the parameter to evaluate the accuracy of the OAAT: it was 0.91 (0.81–1.00) for the diagnosis of OSA (AHI ≥ 5), 0.90 (0.82–0.98) for moderate OSA (AHI ≥ 15), and 0.84 (0.76–0.92) for severe OSA (AHI ≥ 30).* Conclusions. *The OAAT has shown a high correlation with polysomnography and also a high diagnostic accuracy for the diagnosis of OSA. It has also been shown to be able to discriminate among the different degrees of severity of OSA. Additional large studies aiming to validate this questionnaire as a screening or diagnostic test are needed.

## 1. Introduction 

Obstructive Sleep Apnea (OSA) is a type of sleep apnea characterized by repeated episodes of reduction or cessation of airflow during sleep, caused by pharyngeal narrowing or collapse [1].

OSA is associated with an increased risk of morbidity and mortality [2–4] but, once diagnosed, it can be treated effectively with causal therapy (e.g., weight loss), intraoral devices, continuous positive airway pressure (CPAP), and surgery [5]. For these reasons, the main challenge is to identify these patients in order to treat them early.

The gold standard for diagnosis of OSA is polysomnography (PSG). However, the access to PSG is reduced by costs and limited availability [6–9]. Therefore, it is important to identify previously which patients are probably affected by OSA in order to select them for polysomnography. In addition, polysomnography has not a very high sensitivity, and some cases of OSA escape its diagnosis because it is a test of limited duration [10], and because the definitions of apnea, hypopnea, and the Apnea-Hypopnea Index (AHI) cutoff can be considered as human constructs with no physiological objective basis [11].

For these reasons, other diagnostic tests have been proposed as an alternative or in addition to polysomnography, such as respiratory polygraphy through portable monitoring [12, 13], home oximetry [14], some questionnaires (e.g., Berlin Questionnaire, Epworth Sleepness Scale, BANG Questionnaire), and several biologic measurements [15].

When OSA causes excessive daytime sleepiness that is not attributable to other factors and at least 2 other symptoms are present (see American Sleep Disorders Association), there is a syndrome called Obstructive Sleep Apnea Syndrome (OSAS). Since the diagnosis of OSA is often first suspected by the presence of symptoms related to OSAS, Gasparini et al. [10–16] have proposed the use of a questionnaire (Obstructive Airway Child Test (OACT)) for the evaluation of such symptoms in children with craniofacial malformations for the diagnosis of OSA. The OACT showed, in such patients, a good correlation with polysomnography.

The aim of this study was to analyze the diagnostic accuracy of the Obstructive Airway Adult Test (OAAT), a modified OACT questionnaire for adults, compared to polysomnography for the diagnosis of OSA in adult patients.

## 2. Material and Methods 

This study is reported according to the indications of the STARD Statement (STAndards for the Reporting of Diagnostic Accuracy Studies) [17].

### 2.1. Partecipants

Starting from 1st of April 2014, 90 consecutive patients, who arrived to our 5 centers (Department of Maxillo-facial Surgery of the “Catholic University of the Sacred Heart” of Rome (Italy), Department of Otorhinolaryngology and Cervico-Facial Surgery of the “G.B. Morgagni” Hospital of Forlì (Italy), Department of Otorhinolaryngology of the “San Pio X” Rehabilitation Centre of Milan (Italy), Department of Otorhinolaryngology of “Bologna University” (Italy), and Department of Otorhinolaryngology of “V. Fazzi” Hospital of Lecce (Italy)) with a diagnosis of OSA verified with polysomnography not more than one month before (AHI ≥ 5), were recruited to participate to this study. As controls 10 patients (2 for each centre), who were not affected by OSA, were randomly selected and then included into the study. Only patients younger than 18 years old were excluded.

Immediately after the inclusion in the study, one experienced specialist of each centre, who was blind to the polysomnography results, has collected age, sex, height, weight, and BMI of the patients and also interviewed every one of them with the Obstructive Airway Adult Test (OAAT) questions. No treatment was made between polysomnography and the administration of OAAT.

### 2.2. Obstructive Airway Adult Test (OAAT)

The OAAT is composed of 12 multiple-choice questions that include 4 possible answers of decreasing severity. A score, respectively, 10, 8, 4, or 1, was assigned to each answer depending on the severity of the symptom. The OAAT score results from the sum of the scores of every question, so it can vary from a score of 12 up to 120.

As for the OACT, some questions are based on other questionnaires from literature, while other questions have been introduced on the basis of some common symptoms of patients affected by OSA.

The Obstructive Airway Adult Test (OAAT) questions are reported in the appendix.

### 2.3. Overnight Polysomnography

As absolute reference for the comparison of the diagnostic accuracy of the OAAT, we choose the polysomnography because it currently represents the gold standard for the diagnosis of OSA. An apnea-hypopnea index (AHI) greater than 5 per hour of sleep was considered abnormal and was indicative of mild (5 ≥ AHI < 15), moderate (15 ≥ AHI < 30), or severe OSA (AHI ≥ 30) according to Epstein et al. [18].

### 2.4. Statistical Analyses

The continuous variables were analyzed for the normality with the Shapiro-Wilk test. The features of the population in study were summarized for the continuous parametric variables as mean (SD), for the continuous nonparametric variables as median (interquartile ranges 25–75), and for the categorical variables by frequency.

The correlation between OAAT and AHI was calculated using the Spearman rho.

Sensitivity and specificity were used in order to estimate the diagnostic accuracy of the OAAT compared to the polysomnography of, respectively, mild (AHI ≥ 5), moderate (AHI ≥ 15), and severe (AHI ≥ 30) OSA. For each of these possible combinations, we obtained a Receiver Operating Characteristic (ROC) curve, by plotting all sensitivity values on the *y*-axis against their equivalent 1 − specificity values on the *x*-axis for all available thresholds. The area under the ROC curve (95% CI) so determined was evaluated according to the Swets classification [19]:

- AUC = 0.5: the test is not indicative;
- 0.5 < AUC ≤ 0.7: the test is little accurate;
- 0.7 < AUC ≤ 0.9: the test is fairly accurate;
- 0.9 < AUC < 1.0: the test is highly accurate;
- AUC = 1: the test is perfect.

The ideal cutoffs of the OAAT for the diagnosis of mild, moderate, and severe OSA were calculated with the Youden's *S* statistic. The ideal cutoff of the OAAT for the screening of OSA (AHI ≥ 5) as the bigger specificity value has been also detected, among the ones with a 100% sensitivity value.

All statistical analyses were performed using IBM SPSS Statistics v. 21.0 (IBM Corp, Armonk, NY).

## 3. Results 

### 3.1. Participants

A total of 100 patients, 81 men and 19 women, were included in the study from 1st of April 2014 to 14th of May 2014. Their mean age was 52.30 ± 13.06 and their median BMI was 27 (interquartile range 25.00–31.75). The clinical and demographic features of the participants of the study are reported in Table 1.

### 3.2. Polysomnography

The median AHI after polysomnography was 34 (interquartile range 14–49). Ten patients were not affected by OSA (AHI < 5), 16 patients were affected by mild OSA (5 ≤ AHI < 15), 18 patients were affected by moderate OSA (15 ≤ AHI < 30), and 56 patients were affected by severe OSA (AHI ≥ 30).

### 3.3. Diagnostic Accuracy of OAAT

The median OAAT score was 68.50 (interquartile range 46.25–88.00). The Spearman rho test between AHI and OAAT has shown a correlation coefficient of 0.724 (*p* < 0.01).

For the diagnosis of OSA (AHI ≥ 5) the area under the ROC curve for the OAAT was 0.91 (0.81–1.00), which means that the OAAT has been highly accurate for the diagnosis of OSA. The best cutoff for the diagnosis of OSA is OAAT score = 38 (Youden's index 0.74; sensitivity 94%; specificity 80%). The best cutoff for the screening of OSA is OAAT score = 30 (sensitivity 100%; specificity 60%).

For AHI ≥ 15 the area under the ROC curve for the OAAT was 0.90 (0.82–0.98), which means that the OAAT has also been highly accurate in discerning between mild and moderate OSA. The best cutoff in order to discern between mild and moderate OSA is OAAT score = 57 (Youden's index 0.74; sensitivity 89%; specificity 85%).

For AHI ≥ 30 the area under the ROC curve for the OAAT was 0.84 (0.76–0.92), so the OAAT has been fairly accurate in discerning between moderate and severe OSA. The best cutoff in order to discern between moderate and severe OSA is OAAT score = 73 (Youden's index 0.57; sensitivity 68%; specificity 89%).

**Figure 1:**
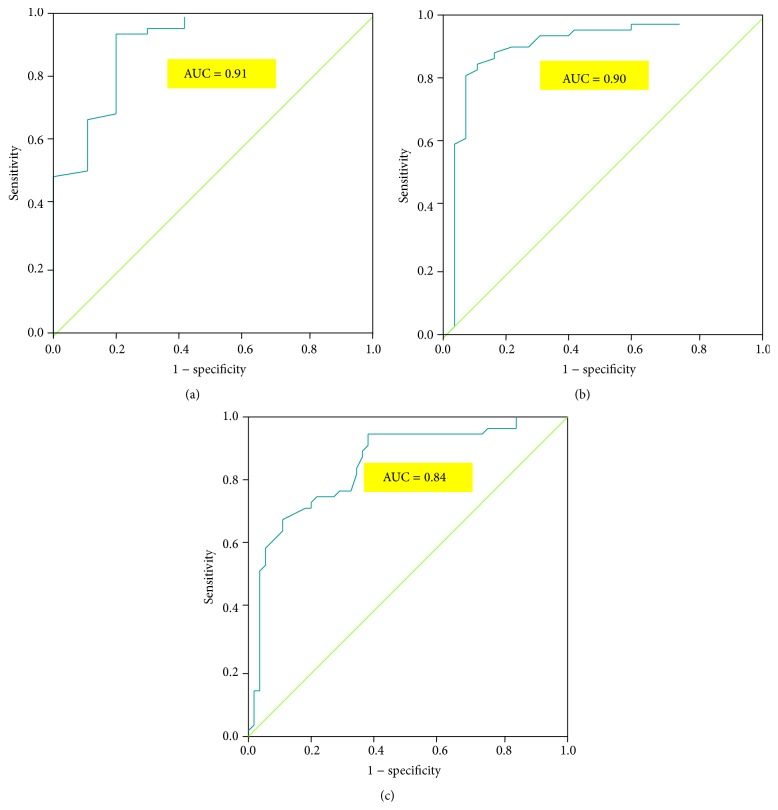
Receiver-operator curves for diagnosis of mild (a), moderate (b), and severe (c) OSA with the Obstructive Airway Adult Test (OAAT).

The Receiver-operator curves for the diagnosis of mild, moderate, and severe OSA by the OAAT are reported in Figure 1; the sensitivity, specificity, AUC, and Spearman rho are reported in Table 2.

## 4. Discussion 

### 4.1. Necessity of New Diagnostic Tests

The ideal screening test of OSA should be inexpensive, simple, and fast and should also have a high sensitivity (ideally of 100%) in order to be able to identify all the cases of disease in a large population and then submit them to a diagnostic test; it should, also, achieve a sufficient specificity in order to avoid unnecessary additional tests.

The ideal diagnostic test of OSA, though inexpensive, simple, and fast, should have both high sensitivity and specificity, in order to discriminate among affected and nonaffected patients.

In the literature, numerous tests and questionnaires have been proposed; they, based on anamnestic, clinical, or biometrical data, have the aim to diagnose or screen OSA in a simpler, faster, and cheaper manner compared to the polysomnography. Among these, the most used ones are the Epworth Sleepiness Scale (ESS) [20], the STOP questionnaire [19], the STOP Bang scoring model [19], and then the Berlin questionnaire [21], but no one of them has a high diagnostic accuracy.

### 4.2. Rationale of the Obstructive Airway Adult Test (OAAT)

The necessity to experiment a new test for the identification of the OSA rises by at least 4 needs.

The first one is to identify (screening) in the general population the patients affected by OSA, because of its high prevalence and its possible consequences on the general health. It could not be actually possible using the polysomnography, because of its high costs and its insufficient accessibility.

The second need is the possibility to control the therapeutic evolution in a simpler, cheaper, and more accessible manner. Performing polysomnography on the same patient many times with the aim of evaluating a therapy could be in several cases a waste of resources: in all this time the accessibility to polysomnography would be, unavoidably, precluded for other patients. In these patients it would be however important to know precisely (for both clinical and research purposes) if the chosen therapy is or is not getting the desired benefits.

The third need is the possibility to diagnose OSA as accurate as possible even in patients who cannot access polysomnography. Different factors could in fact get difficult to perform the polysomnography. The main ones are surely the high costs and the insufficient or null accessibility in least developed countries. Other factors that play a role for the patients nonaffordability to polysomnography are, for example, patients who had not important sleep symptoms, the need to stay one night in the sleep laboratory, and so forth. Considering that this disease and its possible consequences can concern even these groups of people, it would be important to have an alternative test to polysomnography in order to identify them.

In the end, it is known that the polysomnography has not a very high sensitivity, and some cases of OSA escape its diagnosis because it is a test of limited duration [10]. A patient affected by OSA could, in fact, have in the single night of the polysomnography a lesser number of apnea episodes than usual (e.g., because of a pure coincidence or for an alteration of the sleep in a bed he is not used to). Otherwise, submitting all the patients to polysomnography for long times would be very difficult and extremely expensive, so that, a fourth and maybe more ambitious need could be the one to make a diagnostic test able to identify even the hypothetic OSA cases which are not detectable by polysomnography. In this view, a test like the OAAT, based on habitual conditions due to OSA (and not specific for a single episode or a single night), could be very helpful.

### 4.3. OAAT versus Other Diagnostic Tests

According to Pataka et al. [22], for the diagnosis of OSA (AHI ≥ 5) verified with polysomnography, the most used tests are not informative: the AUC of the ESS is 0.42 (0.4–0.46) (sens. 33.3%; spec. 50.6%), the AUC of the Berlin questionnaire is 0.45 (0.4–0.5) (sens. 71.8%; spec. 17.2%), the AUC of the STOP questionnaire is 0.49 (0.45–0.5) (sens. 91.7%; spec. 6.4%), and the AUC of the STOP Bang scoring model is 0.48 (0.4–0.5) (sens. 90%; spec. 4.9%).

Although no direct comparisons have been made, our data state an extremely higher diagnostic accuracy of the OAAT than all the previous tests/questionnaires. In fact, the results of our study show that, for the diagnosis of OSA (AHI ≥ 5), the OAAT is highly accurate (AUC = 0.91 (0.81–1.00)). For an OAAT score <38 (best cutoff found for the diagnosis), the sensitivity was 94% and the specificity was 80%.

If we decided to use the OAAT only as a screening test, the best cutoff would be OAAT score = 30 with a sensitivity of 100% and a specificity of 60%.

In order to discriminate between mild and moderate OSA (AHI ≥ 15), the other tests are little accurate, except the STOP Bang questionnaire which is moderately accurate; in fact, the AUC of the ESS is 0.48 (0.45–0.5) (sens. 44.5%; spec. 52.1%), the AUC of the Berlin questionnaire is 0.48 (0.44–0.5) (sens. 78%; spec. 18%), the AUC of the STOP questionnaire is 0.5 (0.46–0.5) (sens. 92.7%; spec. 6.6%), and the AUC of the STOP Bang scoring model is 0.52 (0.46–0.54) (sens. 94.8%; spec. 5.5%) [22].

Our data show, then, a much higher accuracy of the OAAT compared to all these tests even in the discrimination between mild and moderate OSA. In fact, even for (AHI ≥ 15), the OAAT seems to be highly accurate (AUC = 0.90) (0.82–0.98). For an OAAT score < 57 (best cutoff to discriminate between mild and moderate OSA), the sensitivity was of 89% and the specificity was of 85%.

In order to discriminate between moderate and severe OSA (AHI ≥ 30), the other tests are little accurate, except the STOP Bang questionnaire which results moderately accurate. In fact, the AUC of the ESS is 0.6 (0.57–0.6) (sens. 57%; spec. 62.4%), the AUC of the Berlin questionnaire is 0.6 (0.56–0.6) (sens. 90%; spec. 28.5%), the AUC of the STOP questionnaire is 0.63 (0.6–0.66) (sens. 97%; spec. 11%), and the AUC of the STOP Bang scoring model is 0.72 (0.7–0.75) (sens. 98.7%; spec. 9.9%) [22].

In this case, the accuracy of the OAAT in the discrimination between moderate and severe OSA seems to be moderate, but it still remains considerably higher than the accuracy of all the other tests/questionnaires mostly used. In fact, the AUC was 0.84 (0.76–0.92) and, for an OAAT score <73 (best cutoff to discriminate between moderate and severe OSA), the sensitivity was of 68% and the specificity was of 89%.

In addition to the fact that it has been shown to be able to discriminate among the different degrees of severity of OSA as defined by the polysomnography; in our study the OAAT has also shown a statistically significative correlation with the results of the polysomnography. In other words, as the AHI increases, even the OAAT score increases.

### 4.4. Limitations and Future Researches

This study has some limitations. First of all, this tool has currently only been tested in sleep centres. Patients referred to sleep centers are suspected of having sleep related disorders, especially OSA. So, they are preselected patients. Even if we have included some healthy controls, the OAAT needs to be validated in the other settings with a wider and more representative sample: that could allow for measuring the predictive values and verifying its utility as a screening tool in the general population.

Moreover, the first part of the OAAT is focused on what occurs during the sleep. For these reasons, differences in the perception of these problems could be found among patients that sleep in company or alone. So that it could be interesting, in future studies, to make some subgroup analysis in order to verify such eventuality.

Another potential problem is the fact that the OAAT has been tested for the administration by a doctor and not for the autonomous compilation by the patient himself. Using it in the clinical practice, in fact, several doctors could hypothetically make the questions in a slightly different way, influencing accidentally patients' answers. Therefore the results could be nonrepeatable in the same way for everyone. Moreover even the doctor himself could accidentally make the questions in a different way among different patients. These problems have been partly bypassed because in our study 5 different examinators were present. However, in our opinion, it would be interesting to make some studies in order to measure the level of intra- and interexaminers agreement. Moreover, the results of the administration of this test should be compared with the ones of the self-administration in order to identify the best way to use.

Among the possible applications of the OAAT there are the monitoring of an OSA therapy in progress and the rapid diagnosis of OSA in some emergency situations, when it is important to identify OSA as a risk factor (especially during acute stroke) [23]. However, thug having encouraging results about that, our study did not verify the accuracy in these settings so that additional studies would be useful.

Finally, as said, some OSA cases probably could escape the diagnosis with polysomnography. Because polysomnography is not a test of certainty, valuating the diagnostic accuracy of the OAAT (as any other diagnostic tests) compared to polysomnography could face limitations. Even more so the OAAT could hypothetically identify some cases that polysomnography did not identify. Unfortunately, it is not actually possible to evaluate the accuracy of OSA's diagnostic tests in a different way.

## 5. Conclusions

The OAAT is a cheap, easy, and rapid test for the diagnosis and the screening of OSA by doctors. It has also been shown to be accurate for the diagnosis of mild (OAAT score ≥ 38; AHI ≥ 5), moderate OSA (OAAT score ≥ 57; AHI ≥ 15), and moderately accurate for the diagnosis of severe OSA (OAAT score ≥ 73; AHI ≥ 30).

One strong point seems to be the capacity of the OAAT to discriminate among the different degrees of severity of OSA as actually defined by the polysomnography. That means that there would be no necessity to modify the actual classifying systems and the consequent therapeutic protocols.

The use of this test could be valid for the screening of the general population in order to identify which patient should be submitted to polysomnography, in order to diagnose OSA in patients who cannot access polysomnography and finally to monitor the results of an OSA therapy during time.

A printable version of the test, with its relative scores, is reported in the appendix. Additional large studies aiming to validate this questionnaire as a screening tool in the general population and in order to monitor therapy results are needed.

## Scientific Knowledge on the Subject

The gold standard for the diagnosis of Obstructive Sleep Apnea (OSA) is polysomnography (PSG). The access to PSG is reduced by costs and limited availability, so that additional diagnostic tests are needed.

## What This Study Adds to the Field

The results of this study have shown the high diagnostic accuracy of the Obstructive Airway Adult Test (OAAT), even higher than the accuracy of the other tests available in the literature, for the diagnosis of OSA; moreover, the OAAT seems to be able to discriminate among the different degrees of severity of OSA.

## Figures and Tables

**Table 1:** Demographic and clinic characteristics of 90 adults with Obstructive Sleep Apnea and 10 control subjects.

	OSA (AHI ≥ 5)	Controls (AHI < 5)	Total
	*n* = 90	*n* = 10	*n* = 100
Mean age (±SD)	53.84 (±12.45)	42.00 (±10.20)	52.30 (±13.06)
No. male sex (%)	65 (83.33%)	6 (60.00%)	81 (81.00%)
Median BMI (Interquartile range 25–75)	27.00 (25.00–32.00)	24.50 (22.75–25.25)	27.00 (25.00–31.75)
AHI < 5 (%)	/	10 (10.00%)	10 (10.00%)
5 ≤ AHI < 15 (%)	16 (16.00%)	/	16 (16.00%)
15 ≤ AHI < 30 (%)	18 (18.00%)	/	18 (18.00%)
AHI ≥ 30 (%)	56 (56.00%)	/	56 (56.00%)

**Table 2:** Sensitivity, specificity, Youden's index, and AUC values for diagnosis of mild, moderate, and severe OSA with the Obstructive Airway Adult Test (OAAT).

	Mild OSA (AHI > 5; OAAT score > 38)	Moderate OSA (AHI > 15; OAAT score > 57)	Severe OSA (AHI > 30; OAAT score > 73)
Sensitivity	94%	89%	68%
Specificity	80%	85%	89%
Youden's index	0.74	0.74	0.57
Are under the ROC curve (AUC) (95% C.I.)	0.91 (0.81–1.00)	0.90 (0.82–0.98)	0.84 (0.76–0.92)

**Table 3:** 

Name: ————					
Today's date: ————					
Your age (yrs): ————					
Your sex (male = M; female = F): ————					
#	Questions	10	8	4	1

1	Considering all nights, do you snore?	*⬜* Always	*⬜* Often	*⬜* Sometimes	*⬜* No

2	Your snoring is?	*⬜* Very strong, it can be heard even from another room	*⬜* Very strong	*⬜* Slightly stronger than breath	*⬜* I do not snore

3	Considering a standard night, how long do you snore?	*⬜* All night long	*⬜* Almost all night long	*⬜* Sometimes during a night	*⬜* Never

4	Have your snoring ever woken up other people?	*⬜* Always	*⬜* Often	*⬜* Sometimes during a night	*⬜* Never

5	Have you noticed or someone has reported to you breathing pauses during sleep? If so, how often?	*⬜* Yes, every night with many episodes	*⬜* Yes, many episodes but not every night	*⬜* Sometimes with few episodes	*⬜* Never

6	Do you wake up during the night?	*⬜* Always, even several times during the night	*⬜* Often	*⬜* Sometimes	*⬜* Never

7	Do you have headache or you feel tired in the morning?	*⬜* Always	*⬜* Often	*⬜* Sometimes	*⬜* Never

8	How many airway infections do you have during a year?	*⬜* I'm always ill	*⬜* Very often	*⬜* Sometimes	*⬜* Rarely or never

9	Do you have high blood pressure or current/previous cardiovascular diseases?	*⬜* I have high blood pressure difficultly controllable with medications; in the past I had stroke, cerebral hemorrhages, or cardiac ischemias so that I had to be admitted to hospital	*⬜* I have high blood pressure controllable with medications but in the past I went through ischemic diseases. Actually I take drugs for the therapy of the cardiovascular apparatus and for the control of the arterial pressure	*⬜* I have high blood pressure in pharmacological therapy. I did not go through any cardiovascular event	*⬜* I do not have high blood pressure or cardiovascular diseases

10	Do you have attention deficits at work or while driving?	*⬜* Yes, frequently and I was rebuked; I also had an accident while driving	*⬜* Rarely, but I was rebuked; I also had an accident while driving	*⬜* Rarely I have episodes of drowsiness, but I have not had any accidents while driving	*⬜* Never

11	In your daily life do you ever fall asleep suddenly?	*⬜* Yes. These episodes occurred even when I was driving or talking with someone	*⬜* Yes. These episodes occur even during repetitive activities such as reading newspapers, working on pc, and staying in a waiting room, and so forth	*⬜* Yes. These episodes occur only after lunch even if I drank no alcohol	*⬜* No

12	Do you practice sports that include running or prolonged muscular effort?	*⬜* No, any physical activity is impossible for me because I get tired immediately	*⬜* No, any physical activity is difficult for me because I suffered breathlessness	*⬜* Not much, for laziness or because I have limited time available	*⬜* Yes, always

*Thank you for your cooperation!*					

*For doctors only*

In order to obtain the total OAAT score it is necessary to sum all the scores you made for each one of the 12 questions. OAAT score can vary from 12 up to 120 and can be evaluated according to the following.

*Diagnosis*

*⬜* Mild OSA (OAAT score ≥ 38)

*⬜* Moderate OSA (OAAT score ≥ 57)

*⬜* Severe OSA (OAAT score ≥ 73).

## Appendix

### Obstructive Airway Adult Test (OAAT) Questionnaire and Its Cutoffs for Diagnosis of Mild, Moderate, and Severe OSA

*Obstructive Airway Adult Test (OAAT).* This questionnaire takes only few minutes and it is able to give us information about the eventual presence of a sleeping sickness, not so known but dangerous, called Obstructive Sleep Apnea (OSA). It is important that you answer each question as best you can (Table 3).

## References

[1] Sundaram S., Bridgman S. A., Lim J., Lasserson T. J. (2005). Surgery for obstructive sleep apnoea. *The Cochrane Database of Systematic Reviews*.

[2] Young T., Palta M., Dempsey J., Skatrud J., Weber S., Badr S. (1993). The occurrence of sleep-disordered breathing among middle-aged adults. *The New England Journal of Medicine*.

[3] Young T., Finn L., Peppard P. E. (2008). Sleep disordered breathing and mortality: eighteen-year follow-up of the Wisconsin sleep cohort. *Sleep*.

[4] Nieto F. J., Young T. B., Lind B. K. (2000). Association of sleep-disordered breathing, sleep apnea, and hypertension in a large community-based study. *The Journal of the American Medical Association*.

[5] Myers K. A., Mrkobrada M., Simel D. L. (2013). Does this patient have obstructive sleep apnea? The rational clinical examination systematic review. *The Journal of the American Medical Association*.

[6] Bonnet M., Carley D., Carskadon M. (1992). EEG arousals: scoring rules and examples: a preliminary report from the Sleep Disorders Atlas Task Force of the American Sleep Disorders Association. *Sleep*.

[7] Flemons W. W., Douglas N. J., Kuna S. T., Rodenstein D. O., Wheatley J. (2004). Access to diagnosis and treatment of patients with suspected sleep apnea. *American Journal of Respiratory and Critical Care Medicine*.

[8] Verhulst S. L., Schrauwen N., De Backer W. A., Desager K. N. (2006). First night effect for polysomnographic data in children and adolescents with suspected sleep disordered breathing. *Archives of Disease in Childhood*.

[9] Cheliout-Heraut F., Senny F., Djouadi F., Ouayoun M., Bour F. (2011). Obstructive sleep apnoea syndrome: comparison between polysomnography and portable sleep monitoring based on jaw recordings. *Neurophysiologie Clinique*.

[10] Gasparini G., Di Rocco C., Saponaro G. (2012). Evaluation of obstructive sleep apnea in pediatric patients with facio-craniostenosis: a brief communication. *Child's Nervous System*.

[11] Tam S., Woodson B. T., Rotenberg B. (2014). Outcome measurements in obstructive sleep apnea: beyond the apnea-hypopnea index. *The Laryngoscope*.

[12] Collop N. A., Anderson W. M., Boehlecke B. (2007). Clinical guidelines for the use of unattended portable monitors in the diagnosis of obstructive sleep apnea in adult patients. *Journal of Clinical Sleep Medicine*.

[13] Kimoff R. J. (2011). To treat or not to treat: can a portable monitor reliably guide decision-making in sleep apnea?. *American Journal of Respiratory and Critical Care Medicine*.

[14] Whitelaw W. A., Brant R. F., Flemons W. W. (2005). Clinical usefulness of home oximetry compared with polysomnography for assessment of sleep apnea. *American Journal of Respiratory and Critical Care Medicine*.

[15] Gozal D., Jortani S., Snow A. B. (2009). Two-dimensional differential in-gel electrophoresis proteomic approaches reveal urine candidate biomarkers in pediatric obstructive sleep apnea. *American Journal of Respiratory and Critical Care Medicine*.

[16] Gasparini G., Saponaro G., Rinaldo F. M. D. (2012). Clinical evaluation of obstructive sleep apnea in children. *The Journal of Craniofacial Surgery*.

[17] Bossuyt P. M., Reitsma J. B., Bruns D. E. (2003). Towards complete and accurate reporting of studies of diagnostic accuracy: the STARD initiative. *British Medical Journal*.

[18] Epstein L. J., Kristo D., Strollo P. J. (2009). Clinical guideline for the evaluation, management and long-term care of obstructive sleep apnea in adults. *Journal of Clinical Sleep Medicine*.

[19] Chung F., Yegneswaran B., Liao P. (2008). STOP questionnaire: a tool to screen patients for obstructive sleep apnea. *Anesthesiology*.

[20] Johns M. W. (1991). A new method for measuring daytime sleepiness: the Epworth sleepiness scale. *Sleep*.

[21] Netzer N. C., Stoohs R. A., Netzer C. M., Clark K., Strohl K. P. (1999). Using the Berlin Questionnaire to identify patients at risk for the sleep apnea syndrome. *Annals of Internal Medicine*.

[22] Pataka A., Daskalopoulou E., Kalamaras G., Fekete Passa K., Argyropoulou P. (2014). Evaluation of five different questionnaires for assessing sleep apnea syndrome in a sleep clinic. *Sleep Medicine*.

[23] Camilo M. R., Sander H. H., Eckeli A. L. (2014). SOS score: an optimized score to screen acute stroke patients for obstructive sleep apnea. *Sleep Medicine*.

